# The Double-Edged Sword of Oxidative Stress in Skin Damage and Melanoma: From Physiopathology to Therapeutical Approaches

**DOI:** 10.3390/antiox11040612

**Published:** 2022-03-23

**Authors:** Monica Emanuelli, Davide Sartini, Elisa Molinelli, Roberto Campagna, Valentina Pozzi, Eleonora Salvolini, Oriana Simonetti, Anna Campanati, Annamaria Offidani

**Affiliations:** 1 Department of Clinical Sciences, Polytechnic University of Marche, 60100 Ancona, Italy; m.emanuelli@univpm.it (M.E.); d.sartini@univpm.it (D.S.); v.pozzi@univpm.it (V.P.); e.salvolini@univpm.it (E.S.); 2 New York-Marche Structural Biology Center (NY-MaSBiC), Polytechnic University of Marche, 60131 Ancona, Italy; 3 Department of Clinical and Molecular Sciences, Polytechnic University of Marche, 60100 Ancona, Italy; molinelli.elisa@gmail.com (E.M.); o.simonetti@univpm.it (O.S.); a.campanati@univpm.it (A.C.); a.m.offidani@univpm.it (A.O.)

**Keywords:** oxidative stress, ROS, skin cancer, melanoma

## Abstract

The skin is constantly exposed to exogenous and endogenous sources of reactive oxygen species (ROS). An adequate balance between ROS levels and antioxidant defenses is necessary for the optimal cell and tissue functions, especially for the skin, since it must face additional ROS sources that do not affect other tissues, including UV radiation. Melanocytes are more exposed to oxidative stress than other cells, also due to the melanin production process, which itself contributes to generating ROS. There is an increasing amount of evidence that oxidative stress may play a role in many skin diseases, including melanoma, being the primary cause or being a cofactor that aggravates the primary condition. Indeed, oxidative stress is emerging as another major force involved in all the phases of melanoma development, not only in the arising of the malignancy but also in the progression toward the metastatic phenotype. Furthermore, oxidative stress seems to play a role also in chemoresistance and thus has become a target for therapy. In this review, we discuss the existing knowledge on oxidative stress in the skin, examining sources and defenses, giving particular consideration to melanocytes. Therefore, we focus on the significance of oxidative stress in melanoma, thus analyzing the possibility to exploit the induction of oxidative stress as a therapeutic strategy to improve the effectiveness of therapeutic management of melanoma.

## 1. Introduction

The skin is the largest organ of the human body and represents the primary protection barrier against the external environment. Due to its role, the skin is constantly exposed to oxidative stress from exogenous origins. Among the exogenous sources, the most important are air pollution agents, harmful natural gases such as ozone, exposure to ionizing and non-ionizing radiation, infections from viruses and bacteria, exogenous chemicals, drugs, cosmetics, and toxins [1]. Nonetheless, the skin must also face oxidative stress arising from several endogenous sources, including the classical cell metabolism, the activity of enzymes that can indirectly produce reactive oxygen species (ROS), or the ROS production generated by the neutrophil cells. On the one hand, controlled ROS production is required by the human body, including skin, since ROS function as regulators of the homeostasis in a number of key processes, including epidermal keratinocyte proliferation. On the other hand, ROS are responsible or can worsen skin aging and inflammation, are responsible for DNA, protein, and lipid damage, and thus their toxic activity may lead eventually to skin diseases, including skin cancer [2]. Indeed, in the last decades, numerous systemic pathologies were identified as a consequence of oxidative stress, including cardiovascular disease, cancer, diabetes, rheumatoid arthritis, and neurodegenerative disorders, and consequently, for some of them, it was proposed the use of exogenous antioxidants for prevention or as a treatment strategy [3,4,5,6,7,8]. Therefore, given the dual face of the ROS activity, it appears evident that maintaining a fine balance is crucial for the health, even more for the skin, due to its peculiar higher exposure to oxidative stress sources, such as UV, which does not affect other organs of the body. Despite the fact that the skin owns a large armamentarium of antioxidant defense mechanisms that counteract oxidative stress to prevent its detrimental effects, excessive ROS production cannot always be neutralized. A number of investigations identified variations in the metabolites of ROS or ROS generators and antioxidants in skin diseases, suggesting that an anomalous ROS generation could play a role in their pathogenesis. Pathological conditions in which an involvement of ROS was demonstrated include contact dermatitis [9], urticaria [10], atopic dermatitis [11], psoriasis vulgaris [12], acne vulgaris [13], alopecia areata [14], vitiligo [15] and, above all, skin cancers [16,17]. A particular interest has been raised in elucidating the impact of ROS on melanoma development and progression. Indeed, oxidative stress has emerged as an important player in all stages of melanoma, from genesis to progression until the development of the metastatic disease and chemoresistance, thus being a promising pathway for targeted therapy [18,19]. For instance, a model of redox adaptation has been recently suggested as responsible for developing BRAF inhibitors resistance [20]. Indeed, the use of the BRAF inhibitor vemurafenib for a long period induces the positive selection of resistant melanoma cells that display an augmented ROS production as a consequence of a shift toward a mitochondrial respiration phenotype, together with an increased redox response [20].

Despite the recent efforts focused on this subject, the exact mechanism by which ROS may contribute to melanoma arising progression of aggressiveness are still far to be fully elucidated, and more studies are needed to comprehend the intricate mechanisms by which ROS can exert detrimental effects on normal cell functions in order to elucidate the physiopathology of several skin diseases including, and above all, melanoma [17,18,19,20].

In this review, we have discussed the concept of oxidative stress applied to the skin, summarizing the available literature regarding the interplay between oxidative stress and physiopathology of the skin. Subsequently, we focused on the role of oxidative stress in skin cancers, with particular attention to melanoma, the most aggressive variant of skin cancer, and the perspective of using the double-edged sword of oxidative stress to enhance the efficacy of therapeutics routinely used for the management of skin malignancies.

## 2. Oxidative Stress

The continuous ROS production is sustained by enzymatic activities, including the mitochondrial electron transport chain, nicotinamide adenine dinucleotide phosphate (NADPH), oxidases (NOXs), cyclooxygenases, and xanthine oxidases. However, ROS production can also occur during non-enzymatic reactions, such as the Fenton and Haber–Weiss reactions [21].

The expression “oxidative stress” identifies a condition of imbalance in the tissue between the production of ROS or reactive nitrogen species (RNS) and the capability of the biological system to promptly detoxify these reactive intermediates and/or to repair the following damage. ROS include the free radicals superoxide anion (O_2_^•−^) and hydroxyl radical (^•^OH), and non-radicals as hydrogen peroxide (H_2_O_2_), singlet oxygen (1O_2_) and hypochlorous acid (HOCl), while RNS include nitric oxide (NO^•^), nitrogen dioxide (^•^NO_2_), and peroxynitrite (ONOO^−^) (Table 1) [22].

Both ROS and RNS are continuously produced in living systems, and they are fundamental for the proper functionality of cells and tissues. Indeed, at the cellular level, ROS and RNS are implicated in many processes, which include cell signaling, cellular growth, and apoptosis, whereas at the systemic level participate in complicated functions regulated by the interaction of many factors, such as blood pressure adaptation, cognitive functions and immune response [23]. For instance, during inflammation, the organism generates higher levels of ROS since they exert toxic effects on the pathogens, and in the central nervous system, RNS modulates cerebral blood flow and memory and exerts an important role in sustaining the immune system and the production of cytokines [24,25].

While ROS are mostly produced as a consequence of the normal metabolism of O_2_, RNS arises from the combination of nitric oxide (NO^•^) and ROS. Indeed, while NO^•^ is a relatively inactive free radical, it can react with superoxide anion yielding peroxynitrite (ONOO^−^), a highly RNS, which is responsible for the major part of detrimental cellular effects of RNS [26].

Although accumulation of oxidative injury results in organism death, numerous longevity-promoting mediators enhance ROS generation, which is responsible for activating stress responses that are beneficial for the organism and also prolong the lifespan [27,28]. During an oxidative stress condition, ROS and RNS overproduction overcome the cellular protective mechanisms, turning ROS into detrimental molecules able to damage DNA, proteins, and lipids. ROS and RNS may damage DNA by oxidizing the bases or even triggering single-strand breaks (SSBs) and double-strand breaks (DSBs); all these conditions are highly dangerous since, if DNA is not repaired, it can induce mutagenesis or inhibit replication. These events result in an aberrant gene expression that promotes the transformation toward malignancy [29,30]. Furthermore, ROS can oxidize the lateral chains of amino acids as well as the protein backbone and may induce the formation of disulfide bridges. All these modifications of the classical structure of the protein can result in the impairment of the active sites of the enzymes or in the alteration of the protein’s three-dimensional structure, followed by the degradation of the unfolded or badly folded proteins by the proteasome systems [31]. Finally, in lipid membranes, ROS induce lipid peroxidation, a process characterized by polyunsaturated fatty acid (PUFA) oxidative degradations that result in the formation of lipid radicals and highly reactive aldehydes, which further intensify the toxic impact of free radicals [32]. Similar to ROS, RNS are able to induce damage to proteins, lipids, and DNA, thus exerting detrimental effects on cellular function, which eventually can lead to cellular death. One of the major consequences of elevated RNS levels is PUFA oxidative degradation [33].

The maintaining of a balanced amount of ROS/RNS levels relies on the activity of several enzymes and molecules present in the tissues, which exert a protective effect from oxidative stress damage neutralizing the free radicals, thus called antioxidants. The antioxidant enzymes include the glutathione peroxidases, the superoxide dismutases, the glutathione S-transferases, the catalases, the thioredoxins and thioredoxin peroxidases, peroxiredoxins and heme oxygenase-1, while the antioxidant molecules are represented by glutathione (GSH), several proteins (such as albumin, ferritin, transferrin, ceruloplasmin), scavengers (uric acid, coenzyme Q, and lipoic acid) as well as vitamins (vitamin C, E, and A) [34]. The transcription factor Nrf2 is one of the major players in the modulation of the antioxidant response since it regulates the transcription of several cytoprotective and antioxidant key genes [35]. Indeed, in a condition of oxidative stress, Nrf2 translocates into the nucleus promoting the transcription of the genes encoding for the antioxidant enzymes HO-1, PRDXs, TXN, as well as genes implicated in GSH synthesis [36].

## 3. UV Exposure and Skin Damage

Human skin is constantly exposed to several environmental agents, which are physical or chemical inductors of oxidative stress. Ultraviolet radiation consequent to sun exposure is the most harmful physical factor, being able to damage DNA directly and indirectly. Indeed, UV radiation directly injured DNA, causing the formation of a significant amount of cyclobutane pyrimidine dimers (CPDs), pyrimidine-(6–4)-pyrimidone photoproducts [37]. UVB radiation (280–320 nm) is a major cause of DNA damage in skin cells. Indeed, UVB not only induces the formation of cyclobutane pyrimidine dimers and pyrimidine-pyrimidone (6–4) products but, more importantly, can trigger the formation of SSBs and DSBs on DNA. All these events prevent the transcription and the replication of the DNA, thus impairing the cell functions, and if the damage is not repaired promptly, the inflammation process may occur at the tissue level and even cell death. Nonetheless, errors in DNA repairing can result in the accumulation of mutations, the first step associated with the development of various diseases and skin cancer [38]. On the contrary, small amounts of UVA light (320–400 nm) are beneficial for humans since UVA light is a signal for several photoregulatory proteins involved in restarting circadian rhythms, which themselves modulate numerous different processes in the human body. Nevertheless, if UVA irradiation occurs in high doses, it may weaken the immune system and can induce the formation of ROS through photosensitization reactions [39,40]. This ROS overproduction can damage DNA, inducing the formation of 8-oxo-7,8-dihydro-2′-deoxyguanosine (8-OHdG) that impairs the normal cell functioning and also enhance the expression of matrix metalloproteinases responsible for the degradation of collagen fibers, resulting in wrinkles onset and accelerating skin aging [41,42]. Indeed, although mature melanin displays a specific geometric configuration that exerts a protective function in keratinocytes, it may exert a pro-oxidant effect in melanocytes exposed to UV radiation when it is only partially polymerized. This occurring was suggested to be the possible explanation for the increased risk of melanoma detected in the users of tanning lamps UVA-based [43].

It is widely known that UV irradiation is the main risk factor for all forms of skin cancers, which include melanoma and non-melanoma skin cancers. However, the individual risk of developing a form of skin cancer depends on the ability to promptly repair the DNA, which is negatively correlated with the extension of the damage. Given the protective role of melanin, the sensitivity of skin cells to the serious effects of UV radiation depends largely on the intensity of skin pigmentation. However, the biosynthesis of melanin can be impaired if exposed to several environmental factors, such as UV, and this process has been associated with several dermatological pathologies, including Halo nevi, vitiligo, Vogt-Koyanagi-Harada disease, and malignancy-induced hypopigmentation (from melanoma and mycosis fungoides) [44,45,46,47,48].

## 4. Oxidative Stress in Melanocytes

Melanocytes are cells deriving from the neural crest located in the deepest layer of the epidermis, which exert a key role in protecting the skin from ultraviolet light but recently have been found to play a role also in the immune system. Indeed, one of the fundamental activities of melanocytes is to produce eumelanin and pheomelanin (which are often grouped with the word “melanin”), pigmented molecules that influence skin tone, hair, and eye color [49]. However, several additional activities to photo-protection have been attributed to melanin, such as thermoregulation, antibiotic, cation chelator, free radical sink, and by-product of the scavenging of O_2_^•−^ in the skin [50].

**Figure 1:**
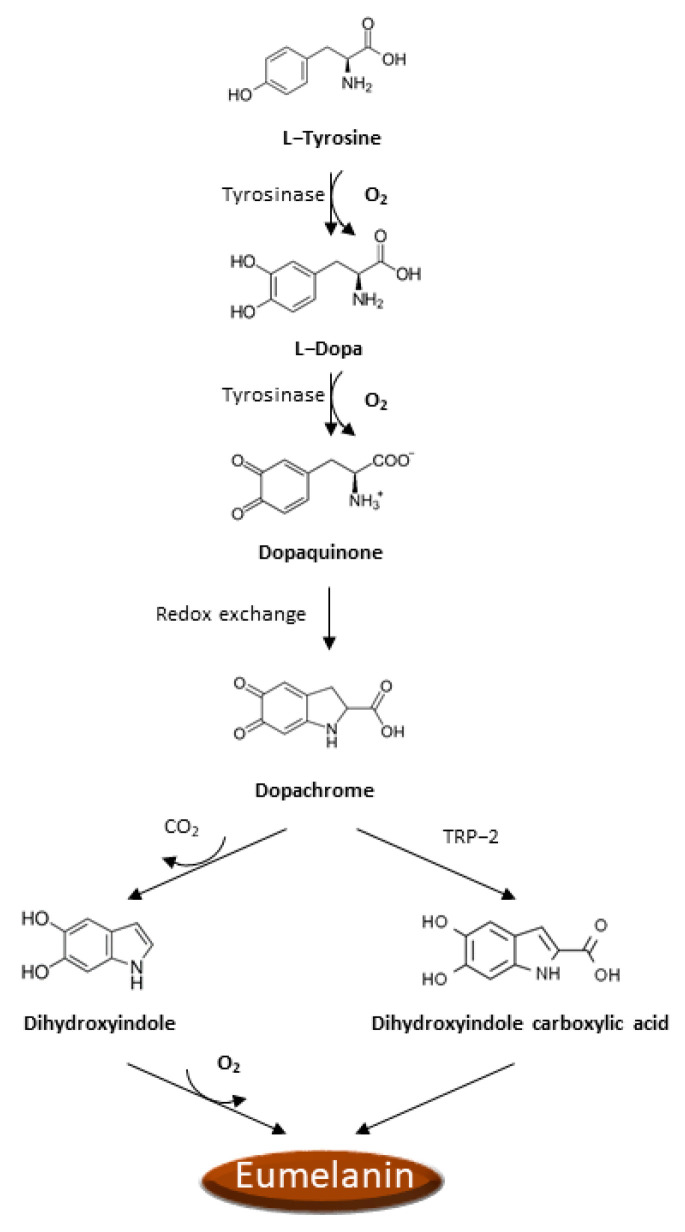
Eumelanin biogenesis pathway.

There is increasing evidence that oxidative stress and ROS formation may promote the melanoma genesis since epidermal melanocytes, from which melanoma arises, are constantly exposed to the ROS production occurring during melanin biosynthesis and to the UVA radiation in addition to all the other sources of ROS production typical of other cells. Analogously to other cancer cells, ROS production is boosted in melanoma cells as a consequence of the activation of numerous recognized oncogenes, inactivation of tumor suppressors [51,52], intratumor hypoxia [50,52], impaired integrin signaling [53], and reprogrammed metabolism [50,54]. Noteworthy, melanoma cells must also face the ROS insult resulting from the process of melanin biosynthesis. Indeed, the process of melanin biosynthesis requires oxidation reactions during which there is a production of O_2_^•−^ and H_2_O_2_ that subjects melanocytes to oxidative stress [55,56]. However, since melanin biosynthesis occurs in the confined cellular compartments named melanosomes, there is some grade of protection of the cellular components from oxidative damage. In the melanin biosynthesis, the enzyme tyrosinase oxidizes tyrosine to L-dopa, which is itself subsequently oxidized to dopaquinone, a reactive molecule toward nucleophilic compounds (e.g., thiols or amino groups), and during these steps, there is the production of O_2_^•−^ [57]. Afterward, a redox exchange converts the dopaquinone into dopachrome, which, after a decarboxylation, which occurs spontaneously, yields dihydroxyindole further oxidized into indole quinone or produces dihydroxyindole carboxylic acid after tautomerization by tyrosinase-related protein 2 (TRP-2), and dihydroxyindole carboxylic acid is next transformed in the related quinone (Figure 1). The TRP-2 exerts a protective effect against oxidative stress since it reduces the harmful effects of quinones and DNA damage induced by free radicals and contextually increases glutathione levels [58]. The process that converts indoles to quinones implicates an important ROS generation [59]. Finally, the polymerization of the quinones results in the formation of black-brown eumelanin. The pheomelanin, which displays a typical red-yellow color, differs from the eumelanin for having a higher ratio of sulfur to quinones, and its biogenesis process has as intermediate the generation of cysteinyl-dopa instead of L-dopa, which is then switched in benzothiazine derivatives. These variations are responsible for the higher pro-oxidant effects caused by the sunlight of pheomelanin with respect to eumelanin.

## 5. Implications of Oxidative Stress in Melanoma

Skin cancers are the most frequent neoplasm diagnosed in the white populations, and their incidence is gradually increasing in the last decade [60]. Skin cancers include malignant melanoma and non-melanoma skin cancers (NMSCs) [61]. Despite the fact that melanoma and NMSCs are different types of malignancies for origin, evolution, and prognosis, they share the two main risk factors, UV exposure and aging [62]. However, among all skin cancers, malignant melanoma is the most aggressive and, although representing only 1% of all skin cancers, it is responsible for the majority of skin cancer-related deaths. It is widely recognized that sunlight induces ROS formation in the skin, impairs the natural antioxidant defenses, and is a major contributor to skin cancer development [63]. Nonetheless, it is noteworthy that aging, another important risk factor for melanoma development, is strictly linked to the accumulation of ROS-induced damages. Thus, the use of antioxidants has been proposed with a chemopreventive aim [64,65,66,67].

Accumulating evidence shows that melanoma displays an anomalous redox state. It has been reported that melanocytes isolated from melanoma patients are characterized by a higher sensitivity to oxidizing molecules that is correlated to an intrinsic antioxidant imbalance, displaying increased intracellular levels of O_2_^•−^ and aberrant activation of the transcription factors AP-1 and NF-κB [68,69,70,71]. It is noteworthy that it was suggested that the redox capacity of melanoma could be reported as a continuum starting from low capacity (typical of normal skin), moderate capacity (a feature of drug-sensitive melanomas), and high capacity (found in drug-insensitive melanomas) [20].

A study that examined which group of genes display an aberrant expression in metastatic melanoma cells revealed that 19 genes involved in the antioxidant response were downregulated, resulting in higher intracellular ROS levels causing dedifferentiation and malignant metastatic progression. Interestingly, in non-metastatic melanoma cells, 10 of these genes were upregulated, suggesting that melanoma late-stage progression is associated with an increased ability of cells to counteract oxidative stress [72].

Few studies demonstrated that melanoma cells display increased levels of enzymes involved in oxidative stress defense, including CAT, SOD, and GSH. In particular, high GSH levels in melanoma cells promote survival to oxidative stress [16,73,74].

Meyskens et al. reported that some transcriptional factors contribute to protecting melanoma cells from oxidative stress damage. Melanoma cells exposed to ROS displayed an augmented activation of AP-1 and NF-kB pathways. Furthermore, superoxide anion levels were found to be upregulated in melanoma compared to melanocytes and were directly correlated with AP-1 expression. Interestingly, the levels of hydrogen peroxide were diminished in melanoma cells and were correlated with NF-kB. Nonetheless, the recruitment of these transcriptional factors did not guarantee control of apoptosis, suggesting a general mechanism by which melanoma cells could escape harmful injury [71].

Yang et al. demonstrated that melanoma cells are characterized by a high expression of neuronal nitric oxide synthase, whose activity produces an abnormal amount of nitric oxide, which was found to be associated with the disease stage. Moreover, the use of selective inhibitors of neuronal nitric oxide synthase induced suppression of melanoma cell proliferation and metastatic capacity [75]. As previously described, melanin synthesis subjects melanocytes to oxidative stress, and therefore, many research efforts were performed in this direction, examining the possibility to establish a connection between melanin biosynthesis, oxidative stress, and melanoma development. Studies focused on dysplastic nevi, which are considered an intermediate between common acquired nevi and melanoma, have demonstrated that they display high ROS, pheomelanin, sulfur, and iron levels, as well as high DNA damage [76,77,78,79]. In hepatocyte growth factor/scatter factor transgenic mice, a model of ultraviolet-induced melanoma, it was demonstrated that frequency of UVA-induced melanoma intensifies with skin pigmentation via an oxidative process involving melanin photo-reactivity whereas, in the same model, tumor initiation is repressed by the antioxidant N-acetylcysteine [66,80]. However, UVA-induced pigmentation does not confer photo-protection, and indoor tanning is correlated with melanoma and non-melanoma skin cancer with a strong association if the first exposure occurred before age 35 year, thus confirming that the use of tanning beds cannot be considered safe and should be avoided [81,82]. Interestingly, it was reported that recessive yellow mice, co-expressing loss-of-function MC1R and activating BRAF^v600E^ mutation are characterized by a significantly higher risk to develop a more invasive melanoma compared to albino ones. Furthermore, pheomelanin synthesis can cause a DNA damage consequent to oxidative stress; therefore, it has been hypothesized that DNA damage resulting from pheomelanin biosynthesis plays a pivotal role in melanoma formation autonomously with respect to UV exposure [82]. However, human melanocytes synthesize eumelanin in addition to pheomelanin, and the balance between the two molecules should influence the impact of UV radiation on the redox state since eumelanin is a ROS scavenger, and its diminution, as occurs in subjects with fair skin, increases the vulnerability of melanocytes to oxidative stress and thus the risk to develop a melanoma [83].

Van der Kemp et al. demonstrated that melanocytes irradiated by UV generate H_2_O_2_ in a dose-dependent fashion coupled with a reduced catalase and HO-1 activity and that UVB irradiation is able to inactivate the OGG1 protein, a key element of the base excision repair (BER) system [84,85,86]. The observation that oxidative stress contributes to the genesis of melanoma is reinforced by the discoveries that mutations in melanoma-associated genes are a consequence of oxidative stress or contribute to worsening the oxidative stress condition. It has been reported that melanocytes are more sensitive to p16 depletion than keratinocytes or fibroblasts, and this depletion can significantly increase ROS levels, thus possibly explaining the association of p16 mutations with melanoma [87]. Furthermore, the loss of PTEN was found to be associated with melanoma progression, apparently due to boosted superoxide anion production consequential to the constant activation of Akt, and the loss of function for the alleles of the gene encoding for the protein MC1R is correlated with an increased risk to develop melanoma due to increased oxidative stress in melanocytes resulting from the failure to respond to α-MSH [88,89]. It was suggested that even the activating ^V600E^BRAF mutation, a somatic mutation frequently expressed in nevi and melanoma, could be the result of a sustained oxidative stress process [90]. Null polymorphisms of two genes belonging to the glutathione S-transferase family of antioxidant genes, GSTM1 and GSTT1, have been correlated with an increased risk to develop melanoma, specifically in subjects with sunburns that occurred in childhood [91]. Furthermore, one single nucleotide polymorphism of the glutathione S-transferase gene GSTP1 that diminishes the enzyme activity has been correlated with melanoma predisposition, with a synergic effect when co-present with alternative alleles of MC1R [92].

**Figure 2:**
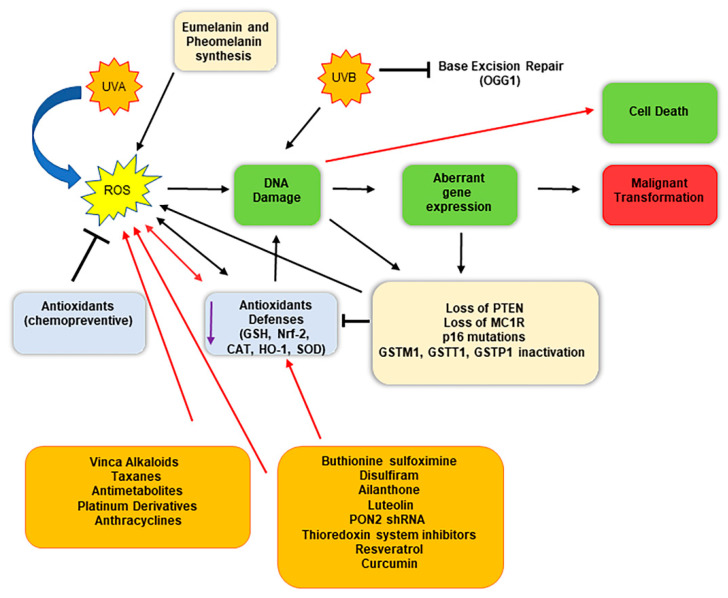
Schematic representation of malignant conversion of melanocytes in melanoma cells following oxidative stress and DNA damage, and therapeutical implications. Melanocytes constantly face oxidative stress due to ultraviolet exposure and melanin biogenesis process, which trigger ROS production. Black arrows indicate the consequential steps following ROS generation; red arrows indicate therapeutical intervention impacts. ROS generation can damage DNA, inducing an aberrant gene expression, eventually triggering malignant transformation. The use of specific molecules (chemotherapeutics and/or compounds that reduce the antioxidant defenses) enhances the detrimental effect of ROS, highly damaging DNA and inducing cell death.

Therefore, taken together, these findings indicate that oxidative stress may be considered as a cofactor able to promote the genesis of melanoma (Figure 2) [65]. Interestingly, high intracellular ROS levels promote not only melanoma genesis and progress but also have been found involved in chemoresistance [93]. The harmful effects of increased intracellular ROS levels are counteracted by melanoma cells through the activity of several redox modulators, which support the antioxidant competence of the cells, including metabolic pathways such as pentose phosphate pathway, lipogenesis, serines biosynthesis, 1-carbon metabolism, and mitochondrial activity.

## 6. Targeting Oxidative Stress Pathways: Therapeutical Implications for Melanoma Management

Due to the higher ROS levels and oxidative stress characterizing cancer cells, it has been hypothesized that the further induction of oxidative stress may lead to killing the cancer cells preferentially. Indeed, despite cancer cells adapting to this condition by developing strong antioxidant mechanisms, they maintain higher ROS levels compared to normal cells [94]. Furthermore, in comparison with other solid tumors, ROS are particularly elevated in melanomas [95]. Therefore, this observation suggests an attractive therapeutic opportunity since neoplastic cells might be more sensitive to drugs that trigger further accumulation of ROS. Currently, a number of chemotherapeutic drugs routinely used are able to induce high levels of oxidative stress. Vinca alkaloids (vincristine, vinblastine, vindesine, and vinorelbine), taxanes (paclitaxel and docetaxel), and antimetabolites (5-Fluorouracil) stimulate the release of cytochrome c from the mitochondria, which itself can induce cell death, and also impair the normal electron transport chain promoting the generation of superoxide anions [96]. However, among the chemotherapeutic drugs, the highest generators of ROS are platinum derivatives (cisplatin, carboplatin, and oxaliplatin) and anthracyclines (adriamycin/doxorubicin, daunorubicin, epirubicin) [97]. The exact mechanism by which these drugs induce ROS is different. For instance, doxorubicin diffuses into the inner membrane of the mitochondria of the myocardium, competing with the coenzyme Q10 in the electron transport chain, thus resulting in a boosted generation of superoxide anions, which is the reason for the cardiotoxicity of this molecule [98]. Instead, the antimetabolite 5-fluorouracil induces the generation of mitochondrial ROS through a p53-dependent pathway [99].

There is increasing evidence that a promising strategy for melanoma treatment might be reducing the antioxidant defenses, such as GSH levels, which are responsible for chemoresistance toward alkylating agents (Table 2). Promising results have been reported coupling chemotherapy with the buthionine sulfoximine (BSO) (Figure 2). BSO is a sulfoximine derivative that reduces levels of GSH by inhibiting the gamma-glutamylcysteine synthetase, the enzyme required in the first step of glutathione synthesis. Indeed, the drug was able to generate a 2.46-fold increase in melphalan cytotoxicity in SK-MEL 28 melanoma cells [100]. Analogously, the use of the drug disulfiram, a potent inhibitor of the enzyme copper-zinc superoxide dismutase, has been reported to enhance the chemosensitivity of melanoma cells to the treatment with oxaliplatin [101,102,103].

Since Nrf2 plays a key role in response to oxidative stress, several compounds that are able to inhibit its activity have been tested. Few studies reported that ailanthone, a vegetable-derived Nrf2 inhibitor, is able to induce oxidative stress and displays antineoplastic effects in several models of chemoresistant tumor cells, including melanoma [104,105]. Brusatol, another Nrf2 inhibitor of vegetable origin, coupled with UVA radiation, repressed melanoma proliferation and significantly augmented intracellular ROS and apoptosis, both in vitro (A375 melanoma cells) and in vivo (heterotopic mouse) models [106]. Another study reported that the Nrf2 inhibitor luteolin can induce glutathione exhaustion in SK-MEL-28 melanoma cells by impairing glutathione S-transferase activity [107].

A promising enzyme that could serve as a therapeutical target in melanoma is the paraoxonase-2 (PON2). PON2 displays anti-oxidative properties following its capability to diminish ROS production, thus counteracting intracellular oxidative stress. Indeed, in mitochondria, the enzyme binds with high affinity to coenzyme Q10 within the inner membrane, thus significantly decreasing the amount of superoxide anion released through the electron transport chain [108]. It was reported that PON2 expression levels are correlated with tumor aggressiveness of several malignancies, including basal cell carcinoma, squamous cell carcinoma, and melanoma [109,110]. Subsequent studies demonstrated that the gene silencing of the enzyme on A375 melanoma cells triggered enhanced chemosensitivity to the cisplatin, and similar results were also obtained in other malignancies, thus suggesting that the enzyme could be an effective target to be coupled with classic chemotherapy [108,111].

Interestingly, cisplatin is not only a chemotherapeutic drug able to induce massive intracellular ROS production, but it also behaves as an inhibitor of the thioredoxin system [112,113]. Indeed, another strategy explored by researchers focused on inhibitors of the thioredoxin system. Thioredoxin cooperates with thioredoxin reductase, thioredoxin peroxidase, and NADPH to detoxify ROS due to its activity to reduce disulfide bonds in proteins owing to the presence of a couple of cysteines located into the active site [114]. It has been reported that the inactivation of thioredoxin triggers oxidative stress, inhibits cell proliferation, and induces cascade signaling impacting kinases that modulate apoptosis [115]. Therefore, several drugs were tested for their inhibitory activity toward the thioredoxin system, including motexafin gadolinium, flavonoids (quercetin), and curcumin [116,117]. Motexafin gadolinium, a porphyrin-like synthetic macrocycle capable of forming highly stable complexes with large metal cations, demonstrated to be able to boost ROS production to toxic levels, with consequent induction of apoptosis through oxidation of thioredoxin, via ASK1-mediated cell death. Additional redox-dependent pathways were also compromised due to the inhibition of the thioredoxin system as a disulfide reductase [113]. The flavonoids myricetin and quercetin were also demonstrated to exert a potent inhibition of thioredoxin reductase [118].

Another approach that provided encouraging results focused on the use of resveratrol, a type of natural phenol that is well known to exert antiproliferative effects. It was demonstrated that resveratrol boosts the radiosensitivity of melanoma cells by inhibiting proliferation and stimulating apoptosis, thus suggesting the use of this molecule as a radiosensitizer for melanoma treatment through radiotherapy [118,119]. Nonetheless, resveratrol showed promising results also when coupled with dacarbazine, a DNA alkylating agent that also stimulates high ROS generation [120,121]. Indeed, Yang et al. demonstrated that resveratrol is able to dock into a druggable pocket of Ref-1 protein, inhibiting its function. Ref-1 is a multifunctional protein involved in DNA base excision repair and redox regulation of many transcription factors, and thus its inhibition by resveratrol significantly enhanced the sensitivity of melanoma cells dacarbazine treatment [120].

Curcumin is a natural molecule whose use was largely investigated in several pathological conditions due to its anti-proliferative, anti-inflammatory, and antioxidant effects [122]. In particular, there is growing evidence that demonstrated that curcumin may promote apoptosis and suppress proliferation in neoplastic cells, including melanoma [123,124,125]. Kocyigit et al. were the first to hypothesize that ROS could play a key role in curcumin-induced DNA damage, apoptosis, and cell death, and thus they examined the effect of curcumin on mouse melanoma cancer cells (B16-F10) and fibroblastic normal cells (L-929). Results obtained demonstrated that curcumin decreased cell viability and mitochondria membrane potential but increased DNA damage and apoptosis at a higher grade in melanoma cells compared to fibroblastic normal cells. Furthermore, all these effects were associated with curcumin-induced elevated ROS production in a dose-dependent fashion [126].

In line with these findings, a subsequent study performed on A375 human melanoma cells has been reported that curcumin administration determined a boosted ROS production, a reduction in glutathione levels, and also disrupted the mitochondria membrane potential with consequent release of cytochrome c from mitochondria to the cytosol triggering the apoptosis cancer cells. Thus, these findings demonstrate that curcumin administration can induce oxidative stress-dependent apoptosis of human melanoma A375 cells, thus suggesting that the pro-oxidant activity of curcumin could be exploited for melanoma treatment [127].

Another interesting study demonstrated that curcumin coupled with tamoxifen, both at low doses, triggered a synergistic stimulation of apoptosis in chemoresistant melanoma cells, an effect that was associated with mitochondria depolarization and ROS generation. Moreover, non-cancerous cells were not affected by the combination of these molecules, thus suggesting a selective cytotoxic effect against melanoma cells [128].

Notably, a study performed by Piskounova et al. reported extraordinary and unexpected conclusions. In this work, it was examined the oxidative stress condition of numerous melanoma disseminating cells and their capacity to induce the formation of metastasis when xenografted into NOD-SCID-Il2rg(-/-) mice. A higher condition of oxidative stress in circulating melanoma cells exerted an inhibitory effect on the formation of distant metastasis. However, contrary to what was expected, if mice were injected with the antioxidant N-acetyl-cysteine, it was detected a significant increase in metastasis formation, while the growth of established sub-cutaneous tumors was not affected, thus generating important questions regarding the use of antioxidants for cancer treatment [129].

In the light of the above-mentioned studies, enhancing the intracellular ROS production by using active biomolecules and/or by targeting enzymes involved in the management of oxidative stress appears to be a promising strategy to potentiate the response of patients to melanoma therapies.

**Table 2:** Summary of studies targeting the oxidative stress pathways in melanoma models.

Molecule	Model	Effect	References
Buthionine sulfoximine	SK-MEL 28 cells	↑ Melphalan cytotoxicity	[101]
Disulfiram	A375, c81-61 cells	↑ Oxaliplatin cytotoxicity	[101]
Disulfiram	A375, c81-46a, c81-61 cells	↓ Proliferation ↑ apoptosis	[102]
Disulfiram	M-14, WM-278, WM-1552c cells	↑ ROS, ↑ apoptosis	[103]
Ailanthone	B16 cells	↑ ROS, ↑ apoptosis	[105]
Brusatol	A375 cells, mouse	↓ Proliferation ↑ ROS, apoptosis	[106]
Luteolin	SK-MEL-28 cells	↓ GSH ↑ ROS	[107]
shRNA PON2	A375 cells	↑ cisplatin cytotoxicity	[108]
Motexafin gadolinium	Recombinant enzyme	↑ ROS, ↑ apoptosis	[116]
Myricetin	Recombinant rat Thioredoxin reductase and cells	↓ Proliferation ↑ ROS	[118]
Quercetin	Recombinant rat Thioredoxin reductase and cells	↓ Proliferation ↑ ROS	[118]
Resveratrol	SK-Mel-5, HTB-65 cells	↑ Radiosensitivity	[119]
Resveratrol	c81-46A, c83-2c cells	↑ Dacarbazine cytotoxicity	[120]
Curcumin	B16, L-929 cells	↓ Proliferation ↑ apoptosis	[126]
Curcumin	A375 cells	↑ ROS, ↑ apoptosis	[127]
Curcumin	A375, G361 cells	↑ Tamoxifen cytotoxicity	[128]

## 7. Conclusions and Remarks

The regulation of ROS and RNS generation has shown involvement in the development of various skin diseases, especially melanoma. To date, the knowledge about the exact mechanism by which ROS and RNS are generated in various conditions, as well as all the machineries implicated in the management of oxidative stress and its impact on melanoma, is far to be fully elucidated. Nevertheless, a number of studies demonstrate that oxidative stress plays a crucial role in melanoma, as a driver of malignant transformation, or as a booster for malignancy progression. This occurring is corroborated by the fact that, in comparison with other solid tumors, ROS are particularly elevated in melanomas. This condition also upregulates RNS, in particular the peroxynitrite, which arises from the combination of anion superoxide and nitric oxide, thus amplifying the detrimental effect of ROS. It is clear that excessive oxidative stress should be avoided in order to reduce the risk of melanoma formation, thus justifying the use of antioxidants as chemopreventive drugs. Indeed, it would be crucial to elucidate the mechanisms underlying the neoplastic transformation in order to be able to design proper strategies to avoid the thorough cell transformation in the neoplastic sense. Nonetheless, oxidative stress could also be considered a powerful tool alley that could be exploited for enhancing the cytotoxicity of therapeutic approaches used for melanoma management. Further studies should be performed to deepen our molecular knowledge of the complex crosstalking pathways involved in the oxidative stress response, in order to design novel specific therapeutic agents that could be used alone or in combination with classic therapies to improve the clinical outcome of melanoma patients.

## Figures and Tables

**Table 1:** Main ROS/RNS radicals and non-radicals present intracellularly.

ROS Radicals	ROS Non-Radicals	RNS Radicals	RNS Non-Radicals
Superoxide anion (O_2_^•−^)	Hydrogen peroxide (H_2_O_2_)	Nitric oxide (NO^•^)	Peroxynitrite (ONOO^−^)
Hydroxyl radical (^•^OH)	Singlet oxygen (1O_2_)	Nitrogen dioxide (^•^NO_2_)	
	Hypochlorous acid (HOCl)		

## Data Availability

The data presented in this study are available in review.
